# 447. Clinical Features, Risk Factors, and Outcomes of COVID-19 in Immunocompromised Adults Hospitalized with Acute Respiratory Infection

**DOI:** 10.1093/ofid/ofad500.517

**Published:** 2023-11-27

**Authors:** Elizabeth G Taylor, Ashley Tippett, Luis W Salazar, Laila Hussaini, Chris Choi, Khalel De Castro, Olivia Reese, Humerazehra Momin, Ashley Lew, Caroline R Ciric, Amrita Banerjee, Amy E Keane, Laura A Puzniak, Robin Hubler, Srinivas Valluri, Timothy L Wiemken, Benjamin Lopman, Nadine Rouphael, Satoshi Kamidani, Evan J Anderson, Christina A Rostad

**Affiliations:** Emory University School of Medicine, Atlanta, Georgia; Emory University, Atlanta, Georgia; Emory University, Atlanta, Georgia; Emory Univeristy, Atlanta, Georgia; Emory University, Atlanta, Georgia; Emory University School of Medicine, Atlanta, Georgia; Emory University, Atlanta, Georgia; Emory University School of Medicine, Atlanta, Georgia; Emory University School of Medicine, Atlanta, Georgia; Emory University, Atlanta, Georgia; Emory University, Atlanta, Georgia; Emory University, Atlanta, Georgia; Pfizer Inc., Collegeville, Pennsylvania; Pfizer Inc., Collegeville, Pennsylvania; Pfizer Inc, New York, New York; Pfizer Inc, New York, New York; Rollins School of Public Health | Emory University, Atlanta, Georgia; Emory University School of Medicine, Atlanta, Georgia; Emory University School of Medicine and Children's Healthcare of Atlanta, Atlanta, Georgia; Moderna, Inc., Atlanta, Georgia; Emory University School of Medicine and Children's Healthcare of Atlanta, Atlanta, Georgia

## Abstract

**Background:**

Individuals with immunocompromising conditions are at high risk of severe disease from COVID-19. The objectives of this study were to describe the clinical features, risk factors, and outcomes of COVID-19 in immunocompromised (IC) adults hospitalized with acute respiratory infection (ARI).

**Methods:**

We enrolled patients ≥ 18 years of age hospitalized with ARI at two Emory University hospitals from May 2021 – Aug 2022. Patient interviews and medical abstractions were completed. Nasopharyngeal and oropharyngeal swabs were tested for SARS-CoV-2 using BioFire Respiratory Panel, and results of standard-of-care testing were recorded. IC was defined using comorbidities from the medical chart (cancer, HIV, organ/stem cell/bone marrow transplant, long-term steroid use, other immunosuppressive conditions). Primary vaccination consisted of 3 mRNA or 1 J&J + 1 other dose for IC patients, and 2 mRNA or 1 J&J for non-IC patients. Vaccine effectiveness (VE) was calculated using a test-negative case-control design. Multivariable logistic regression with stepwise selection yielded a final model controlling for employment, past COVID-19, and blood disorders using SAS v9.4.

**Results:**

Of 1677 enrolled participants, 1653 had SARS-CoV-2 testing, of whom 850 (50.7%) were positive and 231 (27.2% of 850) were IC. Compared to non-IC patients with SARS-CoV-2, IC patients were significantly older (median 58, IQR [44-67)), male (57.1%), and had underlying comorbidities, including blood disorders (13.9%) and chronic kidney disease (36.8%). IC patients were more commonly infected with the Omicron variant, while non-IC patients were more commonly infected with Alpha or Delta. Compared to non-IC, IC patients had longer hospitalization duration (median 4.7, IQR [2.9-9.5]), required positive-pressure ventilation (CPAP/BiPAP) (13.9%), and died (6.5%). IC patients had less commonly received a full COVID-19 vaccine series (19.9% vs. 25.8%) and adjusted VE of primary COVID-19 vaccine series against hospitalization for ARI was lower in the IC (48.7 (17.9, 68.0)) vs. non-IC patients (76.0 (68.4, 81.7)).

**Table 1:**
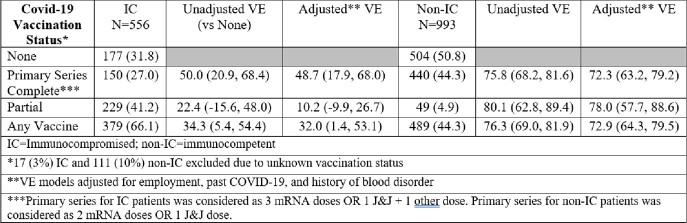
Vaccine Effectiveness in Immunosuppressed vs immunocompetent

**Conclusion:**

Compared to non-IC hospitalized adults, COVID-19 VE against hospitalization for ARI was lower in IC patients, who were more likely to experience severe outcomes and death.

**Disclosures:**

**Laura A. Puzniak, PhD. MPH**, Pfizer, Inc.: Employee|Pfizer, Inc.: Stocks/Bonds **Robin Hubler, MS**, Pfizer, Inc.: Employee|Pfizer, Inc.: Stocks/Bonds **Srinivas Valluri, PhD**, Pfizer Inc: Pfizer Employee and hold Pfizer stocks/options|Pfizer Inc: Ownership Interest|Pfizer Inc: Stocks/Bonds **Timothy L. Wiemken, PhD**, Pfizer Inc: Employee|Pfizer Inc: Stocks/Bonds **Benjamin Lopman, PhD**, Epidemiological Research and Methods, LLC: Advisor/Consultant|Hillevax, Inc: Advisor/Consultant **Nadine Rouphael, MD**, Icon, EMMES, Sanofi, Seqirus, Moderna: Advisor/Consultant **Satoshi Kamidani, MD**, CDC: Grant/Research Support|Emergent BioSolutions: Grant/Research Support|NIH: Grant/Research Support|Pfizer Inc: Grant/Research Support **Evan J. Anderson, MD**, GSK: Advisor/Consultant|GSK: Grant/Research Support|Janssen: Advisor/Consultant|Janssen: Grant/Research Support|Kentucky Bioprocessing, Inc.: Safety Monitoring Board|Moderna: Advisor/Consultant|Moderna: Grant/Research Support|Moderna: Currently an employee|Moderna: Stocks/Bonds|Pfizer: Advisor/Consultant|Pfizer: Grant/Research Support|Sanofi Pasteur: Advisor/Consultant|Sanofi Pasteur: Grant/Research Support|Sanofi Pasteur: Safety Monitoring Board|WCG/ACI Clinical: Data Adjudication Board **Christina A. Rostad, MD**, BioFire Inc.: Grant/Research Support|GlaxoSmithKline Biologicals: Grant/Research Support|Janssen: Grant/Research Support|MedImmune LLC: Grant/Research Support|Meissa Vaccines, Inc.: RSV vaccine technology|Merck & Co., Inc.: Grant/Research Support|Micron Technology, Inc.: Grant/Research Support|Moderna, Inc.: Grant/Research Support|Novavax: Grant/Research Support|PaxVax: Grant/Research Support|Pfizer, Inc.: Grant/Research Support|Regeneron: Grant/Research Support|Sanofi Pasteur: Grant/Research Support

